# The Incorporation of Nanoconfined Poly(ionic liquid)s with Two-Dimensional Covalent Organic Frameworks to Enhance Proton Conduction

**DOI:** 10.3390/molecules30051004

**Published:** 2025-02-21

**Authors:** Yonghong Wang, Xiaoxiao Liang, Ming Wang, Jiahui Wang, Yanan Gao, Fei Lu

**Affiliations:** Key Laboratory of Ministry of Education for Advanced Materials in Tropical Island Resources, School of Chemistry and Chemical Engineering, Hainan University, Haikou 570228, Chinawangjh@hainanu.edu.cn (J.W.)

**Keywords:** poly(ionic liquid)s, covalent organic frameworks, proton conduction, nanoconfinement, composite membranes

## Abstract

Covalent organic frameworks (COFs) hold promising potential as high-temperature proton conductors due to their highly ordered nanostructures and high specific surface areas. However, due to their limited functional groups and poor membrane-engineering properties, finding practical applications for COF-based proton-conducting materials still remains challenging. Herein, we proposed a universal strategy to fabricate proton-conducting composite membranes by the incorporation of sulfonic acid-bearing COFs and zwitterionic poly(ionic liquid)s (PILs) via in situ polymerization. Zwitterionic PILs with methanesulfonate counter ions can work as the intrinsic proton sources, and the sulfonic acid groups on the COF nanochannels can act as the extrinsic proton suppliers. Benefiting from the spatial nanoconfinement of long-range ordered nanochannels and the enhanced electrostatic interactions with PILs, the COFs with high densities of sulfonic acid groups can endow the as-prepared composite membrane (PIL@TpBD(SO_3_H)_2_) with a comparable anhydrous proton conductivity of 3.20 × 10^−3^ S cm^−1^ at 90 °C, which is much higher than that of conventional Nafion (~10^−5^ S cm^−1^ at 90 °C under anhydrous condition). ^1^H NMR DOSY spectra reveal that both the diffusion and dissociation of protons can be drastically facilitated upon nanoconfinement, demonstrating the promising efficiency of nanochannels in proton conduction. Moreover, the obtained composite membranes possess outstanding mechanical and thermal stability, which is crucial for their practical application. This study demonstrates proton conduction elevation in nanoconfined PILs and provides a promising insight into the engineering of stable COF-based proton-conducting materials.

## 1. Introduction

The proton exchange membrane fuel cell (PEMFC) is an environmentally friendly energy conversion device that has garnered increasing attention in recent years due to its high power density, good stability, and easy start-up [1,2]. As the central element, the proton conductivity of its proton exchange membrane (PEM) plays a vital role in the high efficiency of the PEMFC [3,4]. Currently, the perfluorosulfonated polymer, Nafion, with a hydrophobic polytetrafluoroethylene backbone and a hydrophilic sulfonic acid side chain, has been widely applied in industry, owing to its high conductivity of ~0.1 S cm^−1^ at 80 °C with 100% humidity [5,6]. The performance of Nafion would be severely impeded with an increased working temperature and reduced humidity [7]. In this regard, there is a strong need for PEMs to work in high-temperature and low-humidity conditions, which are favorable for accelerating electrochemical reactions and improving the CO tolerance of metal catalysts [8]. Thus, it is of significance to develop proton-conducting materials with high-performance at elevated temperatures and limited humidity.

Recently, crystalline porous materials with built-in ordered nanochannels have shown remarkable advantages in ion transport [9,10,11,12,13]. Specifically, two-dimensional (2D) covalent organic frameworks (COFs) have emerged as part of the new field of proton-conducting materials due to their structural tunability, ordered one-dimensional (1D) nanoporous channels, and excellent chemical stability in the presence or absence of water [14,15,16,17]. However, most COFs always lack mobile protons. Introducing acidic groups (such as –SO_3_H) onto the framework or filling the pores with low-pKa molecules (such as H_3_PO_4_, triazole, or imidazole) can form proton conduction pathways [18,19,20]. Although great achievements have been made, the majority of proton-conducting COF materials have to be compressed into pellets with very limited mechanical strength due to their rigid powder state, which hampers their large-scale practical application [21,22,23]. Given this, the introduction of COFs as nanofillers into polymeric matrixes to fabricate composite membranes provides a promising solution to improve the processability of COFs. For instance, the incorporation of phosphoric acid-loaded COFs into a Nafion matrix has been developed to produce composite proton-conducting membranes [24]. The formed nanochannels for proton conduction in these composite membranes always show a relatively high dependence on the chemical structure and interactions between the matrix and COFs. Thus, developing novel materials to fabricate COF-based membranes with efficient proton conduction and stable mechanical properties is highly desired.

Ionic liquids (ILs) with a low volatility, high ionic conductivity, wide electrochemical window, and high thermal and chemical stability are ideal candidates for high-temperature ionic conductors [25,26,27,28]. Combining ILs with polymer backbones gives advantages in terms of a higher operating temperature range and improved processability. From this starting point, poly(ionic liquid)s (PILs), which are prepared by the polymerization of ionic liquid monomers, can integrate the promising characteristics of ILs into polymeric matrixes, and have been reported to work as high-temperature PEMs [29,30,31]. However, the intrinsic high ionic conductivity of ILs or PILs is mainly dominated by their component ions, which may be irrelevant to the electrochemical reactions. It is still a big challenge to introduce target ions (such as protons for fuel cells) into IL/PIL-based proton-conducting materials. To address this issue, zwitterions, in which cations and anions are intramolecularly linked, have been proposed as a potential strategy to prepare proton-containing PILs by the equimolar mixture of zwitterions and acid [32]. In addition, the zwitterionic structure can also facilitate proton hopping from the Brønsted acid to the base [33,34]. And the molecular-level size of PIL monomers can make them ideal for impregnating into the pores of COFs to fabricate nanoconfined proton transporters [35,36]. The use of zwitterionic PILs as intrinsic proton sources is an important breakthrough in the field of proton-conducting materials. Unlike conventional PILs that rely on external proton carriers (e.g., water, acid) or ionic liquids (ILs), zwitterionic PILs inherently contain proton-donor groups (e.g., -SO_3_H, -PO_3_H_2_). This unique design achieves humidity-independent proton conduction by its built-in protons. Moreover, the hopping mechanism (proton hopping via a hydrogen bonding network) and the vehicle mechanism (via the migration of protons) synergistically enhance the overall electrical conductivity. This paradigm can overcome a key limitation of traditional PIL-based materials and is particularly applicable to fuel cells that require stable operation in complex environments [37].

PIL@COF composite membranes stand out by synergistically integrating the unique advantages of PILs and COFs. PILs provide the material with a high ionic conductivity and flexibility, while COFs offer a highly crystalline, ordered porous structure and exceptional thermal/chemical stability. This composite strategy enables precise proton transport through the regular channels of the COFs, maintaining a high conductivity even under low humidity. Compared to Nafion, PIL@COF membranes operate efficiently across a broader temperature range, owing to the thermal stability of COFs up to 400 °C, and in low-humidity conditions. Additionally, the mechanical strength derived from the rigid framework of COFs can effectively mitigate the swelling and durability challenges observed in traditional polymer membranes.

**Scheme 1:**
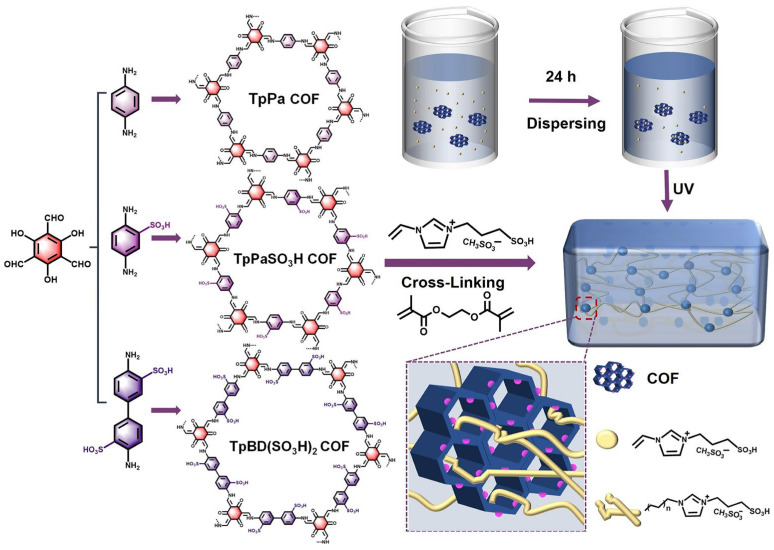
Synthesis of TpPa COF, TpPaSO_3_H COF, and TpBD(SO_3_H)_2_ COF, and schematic diagram of preparation of PIL@COF composite membranes.

Based on these considerations, here we constructed COFs with various grafting densities of grafting sulfonic acid (–SO_3_H) through Schiff-base condensation reactions between the aldehyde monomer 1,3,5-triformylphloroglucinol (TFP) and three different amine monomers: p-phenylenediamine, 2,5-diaminobenzenesulfonic acid, and 4,4′-diaminodiphenyl ether-3,3′-disulfonic acid, to give TpPa COF, TpPaSO_3_H COF, and TpBD(SO_3_H)_2_ COF, respectively (Scheme 1). Furthermore, the proton-containing zwitterionic PIL monomer 1-vinyl-3-(3-sulfopropyl)-imidazolium methanesulfonate was confined into the 1D nanochannels of the COFs and in situ cross-linked to fabricate PIL@COF composite membranes (donating as PIL@TpPa, PIL@TpPaSO_3_H, and PIL@TpBD(SO_3_H)_2_). The zwitterionic PIL with methanesulfonate counter ions could work as the intrinsic proton donors and the sulfonic acid groups on COF nanochannels could act as the extrinsic proton donors. Benefiting from the spatial nanoconfinement of the long-range ordered nanochannels and its enhanced electrostatic interactions with PIL, the as-prepared composite membrane PIL@TpBD(SO_3_H)_2_ exhibited a comparable proton conductivity of 3.20 × 10^−3^ S cm^−1^ at 90 °C, as well as outstanding mechanical and thermal stability. This work will showcase the capabilities of COFs in materials for proton conduction and broaden the range of applications for PILs.

## 2. Results and Discussion

### 2.1. Preparation and Characterization of PIL@COF Composite Membranes

The COFs with various grafting densities of grafting sulfonic acid (–SO_3_H) were constructed by Schiff-base condensation reactions between the aldehyde monomer 1,3,5-triformylphloroglucinol (TFP) and three different amine monomers—p-phenylenediamine, 2,5-diaminobenzenesulfonic acid, and BD(SO_3_H)_2_—to give TpPa COF, TpPaSO_3_H COF, and TpBD(SO_3_H)_2_ COF, respectively [38]. Furthermore, the proton-containing PIL monomer 1-vinyl-3-(3-sulfopropyl)-imidazolium methanesulfonate was added and thoroughly mixed with each of the three COFs to ensure the diffusion and impregnation of the PIL monomer into the 1D nanochannels of the COFs. Then, the well-defined proton-conducting membranes were formed via in situ cross-linking polymerization (donating as PIL@TpPa, PIL@TpPaSO_3_H, and PIL@TpBD(SO_3_H)_2_, respectively). Figure S3 displays the photographs of the pure PIL membrane and PIL@COF membranes, in which all the PIL@COF membranes exhibited an obvious deepening in color compared to that of the PIL membrane.

**Figure 1:**
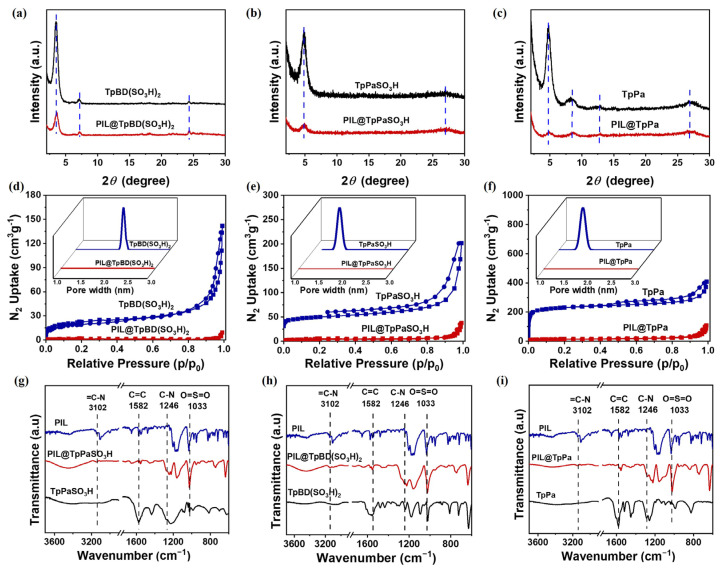
Characterization of COFs and PIL@COFs. XRD patterns of as-synthesized (**a**) TpBD(SO_3_H)_2_ and PIL@TpBD(SO_3_H)_2_; (**b**) TpPaSO_3_H and PIL@TpPaSO_3_H; (**c**) TpPa and PIL@TpPa. BET specific surface area and pore size distribution of (**d**) TpBD(SO_3_H)_2_ and PIL@TpBD(SO_3_H)_2_; (**e**) TpPaSO_3_H and PIL@TpPaSO_3_H; (**f**) TpPa and PIL@TpPa. FT-IR spectra of as-synthesized (**g**) PIL@TpBD(SO_3_H)_2_; (**h**) PIL@TpPaSO_3_H; (**i**) PIL@TpPa.

The microstructures and chemical structures of the COFs and PIL@COFs were investigated by detailed characterizations. Firstly, the crystalline structures of the as-synthesized COFs were examined by powder X-ray diffraction (PXRD). The PXRD pattern of the TpBD(SO_3_H)_2_ COF (Figure 1a) exhibited a strong peak at 3.56°, together with two prominent peaks at 7.06° and 24.34°, which corresponded to the (100), (110), and (001) facets, respectively. For the TpPaSO_3_H COF (Figure 1b), a sharp diffraction peak at 4.7° and a small peak at 26.8° were assigned to the reflections from the (100) and (001) facets, while the TpPa COF exhibited four obvious diffraction peaks at 4.7°, 8.3°, 12.7°, and 26.7°, which can be ascribed related to (100), (110), (200), and (001) facets, respectively (Figure 1c). The diffraction peaks of all three COFs were consistent with the simulated eclipsed AA-stacking models [39,40]. After incorporation with the PIL monomers, the obtained PIL@COFs showed very similar diffraction peaks with a decrease in peak intensity, indicating the occupation of the nanopores of the COFs by the PIL monomers (Figure 1a–c).

Additionally, N_2_ adsorption/desorption isotherms were conducted at 77 K to evaluate the surface area and permanent porosity of the COFs and PIL@COFs. All the N_2_ desorption isotherms of the three COFs in Figure 1d–f are Type IV adsorption isotherms with a hysteresis loop, indicating their mesoporous characteristics. The BET specific surface areas of the TpBD(SO_3_H)_2_, TpPaSO_3_H, and TpPa COFs were calculated to be 78.0, 101.0, and 850.0 m^2^ g^−1^, respectively. Their corresponding average pore diameters were estimated to be 2.1, 1.5, and 1.7 nm. Upon the incorporation of PIL into the nanochannels of the COFs, the specific surface areas of PIL@TpBD(SO_3_H)_2_, PIL@TpPaSO_3_H, and PIL@TpPa decreased to 17.9, 3.0, and 50.0 m^2^ g^−1^, and the pores of the PIL@COFs vanished, demonstrating that the COF nanochannels were filled with PIL [41]. Furthermore, differential scanning calorimetry (DSC) measurements were also carried out to detect the possible phase transition of the confined PIL. As shown in Figure S4, the bulk PIL monomers showed two sharp peaks at 74 °C and 188 °C, and a broad peak around 100 °C, which could be ascribed to the melting and cross-linking behaviors of the PIL and the evaporation of moisture, respectively, while after the confinement of the PIL into the COFs, no peaks for the phase transition of the PIL could be observed (only the evaporation of moisture was retained), confirming that the PIL monomers confined into the COF nanopores were remarkably solidified.

Fourier transform infrared spectroscopy (FT-IR) analysis was utilized to determine the chemical compositions of the PIL, COFs, and PIL@COFs. As shown in Figure S6, the peak at 1637 cm^−1^ for the C=O band in the aldehyde monomers and the peak at 3300–3425 cm^−1^ for the N-H band in the amine monomers disappeared, demonstrating the complete condensation reaction between the aldehyde and amine monomers. Meanwhile, as shown in Figure 1g–i, the new peaks at 1582 cm^−1^ for the C=C band and the absence of peaks for the C=N band revealed an irreversible enol–keto tautomerization, which enhanced the structural stability of the COFs [42]. Additionally, the characteristic peaks at 1033 cm^−1^ for the O=S=O band were also displayed in the FT-IR spectra of the TpBD(SO_3_H)_2_ and TpPaSO_3_H COFs. Upon incorporation with PIL, a new peak at 3102 cm^−1^ for the =C-N bond in the imidazolium cation was observed for all PIL@COFs, confirming the successful impregnation of the PIL. It is worth noting that the O=S=O peak intensity in the PIL@COFs was significantly stronger than that in TpBD(SO_3_H)_2_ and TpPaSO_3_H, suggesting that the strong electrostatic interactions between the –SO_3_H groups of the COFs and the zwitterionic PIL facilitated the dissociation of protons in –SO_3_H groups.

**Figure 2:**
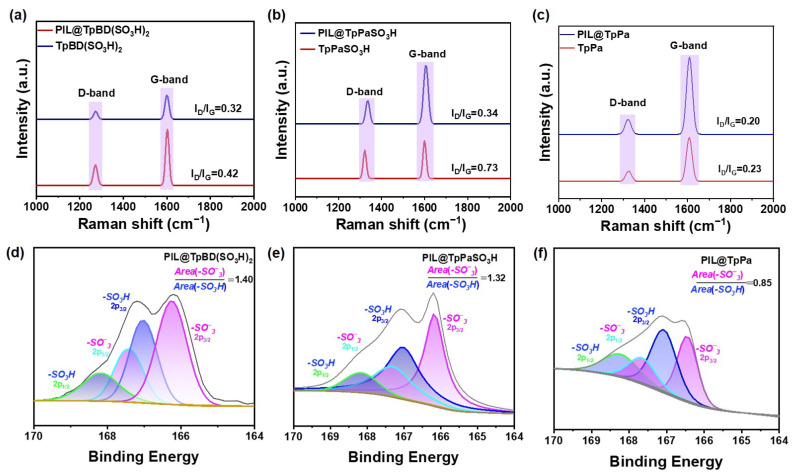
Raman spectra of as-synthesized (**a**) TpBD (SO_3_H)_2_ and PIL@TpBD (SO_3_H)_2_; (**b**) TpPaSO_3_H and PIL@TpPaSO_3_H; (**c**) TpPa and PIL@TpPa. XPS spectra of S 2p for as-synthesized (**d**) PIL@TpBD(SO_3_H)_2_; (**e**) PIL@TpPaSO_3_H; (**f**) PIL@TpPa.

The interactions between the COFs and PIL, especially the electrostatic interactions between the –SO_3_H groups on the COF nanochannels and the zwitterionic headgroups of the PIL, were further evaluated by Raman spectra. As illustrated in Figure 2a–c, all COFs and PIL@COFs exhibited a D band near 1277 cm^−1^ and a G band near 1613 cm^−1^ [43]. The intensity ratios of the D and G bands (ID/IG) for TpBD(SO_3_H)_2_, TpPaSO_3_H, and TpPa were 0.32, 0.34, and 0.20, respectively. After the incorporation of PIL, the *I*_D_/*I*_G_ values for PIL@TpBD(SO_3_H)_2_ and PIL@TpPaSO_3_H were increased to 0.42 and 0.73, indicating a reduction in the symmetrical structure of the corresponding PIL@COF due to the strong electrostatic interactions between the –SO_3_H groups of the COFs and the zwitterionic groups in the PIL. In contrast, the *I*_D_/*I*_G_ value for PIL@TpPa was 0.23, close to that of the TpPa COF (0.20), suggesting that the weak interactions between the PIL and the TpPa COF were due to the lack of –SO_3_H groups on the COF nanochannels. Additionally, X-ray photoelectron spectroscopy (XPS) was also utilized to explore the interactions between the above-mentioned groups. For the materials which have two sulfur species, the S 2p spectra can be deconvoluted into four peaks assigned to two groups of S 2p3/2-S 2p1/2 spin–orbit splitting. And the total peak area for each sulfur species can be related to the concentration of each group [41]. As shown in Figure S7, for the pure PIL, the two peaks at 167.4 eV and 168.6 eV are assigned to the 2p3/2 and 2p1/2 of the sulfur originating from the –SO_3_H group, while the two peaks at 166.6 eV and 167.8 eV are assigned to the 2p3/2 and 2p1/2 of the sulfur originating from the –SO_3_^−^ group due to its electronegativity. For the TpPa COF, there was no typical peak for S 2p due to the lack of an –SO_3_H group (Figure S8c), while for PIL@TpPa, the emergence of peaks for S 2p further indicated the successful incorporation of the PIL into the COF (Figure S8f). These two similar groups of S 2p3/2-S 2p1/2 spin–orbit splitting can also be observed in the S 2p spectra of PIL@TpBD(SO_3_H)_2_ and PIL@TpPaSO_3_H. Due to the weak interactions between the PIL and the TpPa COF, the area ratios of –SO_3_H and –SO_3_^−^ for PIL@TpPa was 0.85 (Figure 2f), similar to that of pure PIL. This revealed that the TpPa COF had a negligible influence on the dissociation of protons in the PIL. In contrast, the area ratios for PIL@TpBD(SO_3_H)_2_ and PIL@TpPaSO_3_H were increased to 1.40 and 1.32 (Figure 2d–e), suggesting that the increased proportion of –SO_3_^−^ groups in these two PIL@COF composite membranes. We proposed that the strong electrostatic interactions between the –SO_3_H groups of the COFs and the zwitterionic headgroups of the PIL promote the dissociation of –SO_3_H groups, resulting in the increase of –SO_3_^−^ and free protons.

### 2.2. Morphology, Mechanical Properties, and Thermal Stability of PIL@COF Composite Membranes

**Figure 3:**
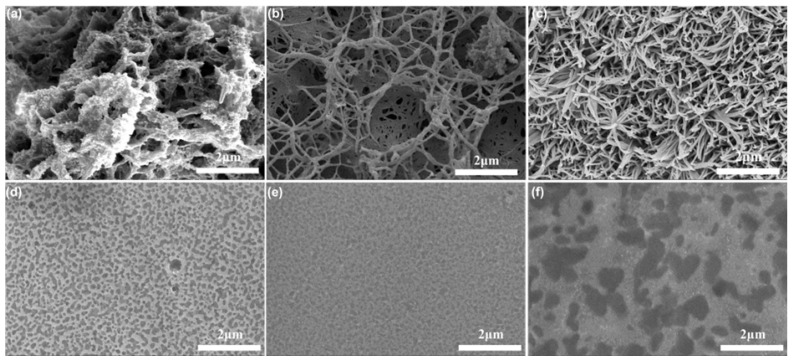
SEM surface images of COFs and PIL@COF composite membranes. (**a**) TpBD(SO_3_H)_2_; (**b**) TpPaSO_3_H; (**c**) TpPa COF; (**d**) PIL@TpBD(SO_3_H)_2_; (**e**) PIL@TpPaSO_3_H; (**f**) PIL@TpPa.

Scanning electron microscopy (SEM) was used to observe the morphologies of the prepared COFs and PIL@COFs. Figure 3a–c display the surface SEM images of TpBD(SO_3_H)_2_, TpPaSO_3_H, and TpPa COF, revealing complex and rough microstructures with densely entangled networks similar to fibrous structures. After incorporating the PIL, more flat and dense structures can be observed for the PIL@COF composite membranes (Figure 3d,e). We proposed that the interactions between the COFs and PIL would effectively confine PIL monomers within the 1D nanopores of the COFs, resulting in continuous and dense structures through in situ polymerization [35,36]. In addition, the COFs and PIL@COFs were examined via energy-dispersive X-ray spectroscopy (EDS) mapping (Figures S9–S11). It can be observed that the PIL was uniformly dispersed throughout the entire structure of COFs.

**Figure 4:**
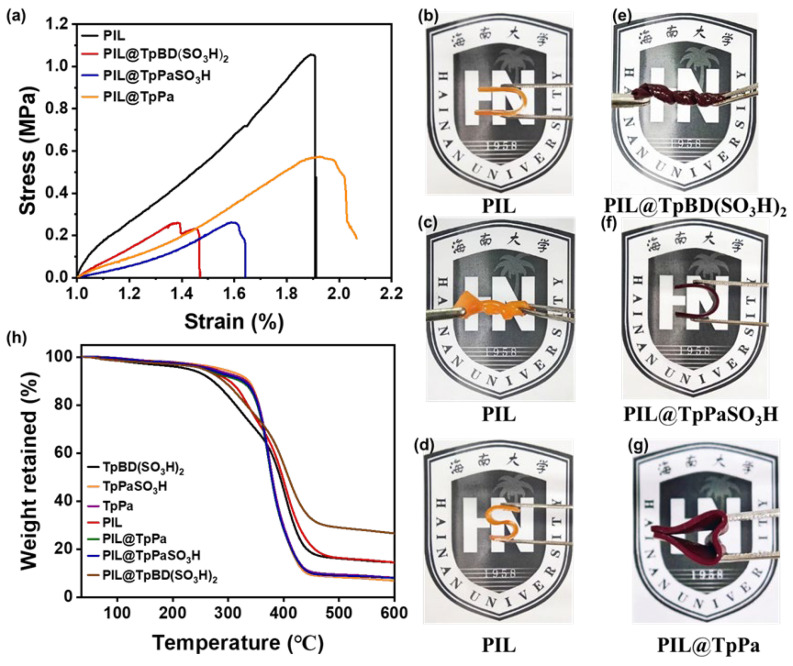
(**a**) Mechanical properties of PIL and PIL@COF composite membranes. (**b**–**g**) Photographs of highly flexible PIL and PIL@COF composite membranes. (**h**) TGA curves of COFs, PIL, and PIL@COFs.

The mechanical properties of PIL@COF composite membranes are presented in Figure 4a. Although the doping of the COF nanoparticles into the PIL matrix resulted in a slight loss in mechanical properties, all the PIL@COF composite membranes exhibited a comparable tensile strength and good flexibility. As demonstrated in Figure 4b–g, the PIL and PIL@TpBD(SO_3_H)_2_ membranes can be freely twisted, while the PIL@TpPaSO_3_H and PIL@TpPa membranes can be folded into “U” and “S” shapes. Additionally, all the membranes were capable of self-healing, returning to their original rectangular shape as shown in Figure S12. This was attributed to the physical entanglement and electrostatic interactions formed between the PIL and COFs [44], which in turn ensured that the composite membranes doped with rigid COF powder maintained good mechanical properties [45,46]. The thermal stability of the COFs, as well as the PIL and PIL@COF composite membranes, was also investigated. The TGA curves in Figure 4h demonstrate that the prepared composite membranes are thermally stable up to 250 °C, which enables their prospective application in fuel cells.

### 2.3. Proton Conduction Properties of PIL@COF Composite Membranes

**Figure 5:**
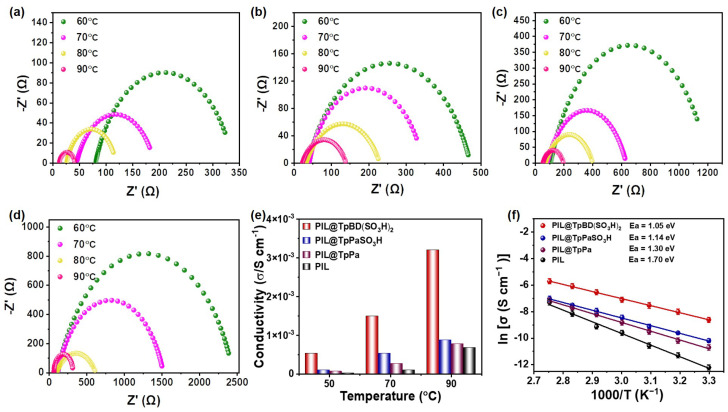
Nyquist plots of membranes at different temperatures for (**a**) PIL@TpBD(SO_3_H)_2_; (**b**) PIL@TpPaSO_3_H; (**c**) PIL@TpPa; (**d**) PIL. (**e**) Conductivities of PIL and PIL@COFs at 50 °C, 70 °C, and 90 °C. (**f**) Arrhenius plots for PIL and PIL@COFs.

By employing an in situ polymerization method, a free-standing pure PIL membrane and PIL@COF composite membranes with diameters of 1 cm and thicknesses of 1 mm were prepared, which were then assembled into corn cells with stainless steel electrodes. For comparison, pristine COF powders were also milled and pressed into self-supporting pellets to be assembled into corn cells. Electrochemical impedance spectroscopy (EIS) analysis was conducted within the temperature range of 30 to 90 °C to determine their temperature-dependent proton conductivity. As shown in Figure 5a–d, the Nyquist plots for the pure PIL and all three PIL@COF composite membranes displayed a significant decrease in impedance with increasing temperature. For the pure PIL membranes, the proton conductivity was 7.46 × 10^−5^ S cm^−1^ at 50 °C, which gradually increased to 6.80 × 10^−4^ S cm^−1^ as the temperature was raised to 90 °C (Figure 5e). As shown in Figure S13, all the COFs exhibited a relatively low conductivity at 30 °C, which was 1.91 × 10^−8^ S cm^−1^ for the TpBD(SO_3_H)_2_ COF, 1.97 × 10^−10^ S cm^−1^ for the TpPaSO_3_H COF, and 2.58 × 10^−11^ S cm^−1^ for the TpPa COF, respectively. Upon the temperature being increased to 90 °C, the conductivities of the COFs only exhibited a limited enhancement, i.e., 7.65 × 10^−8^ S cm^−1^ for the TpBD(SO_3_H)_2_ COF, 7.93 × 10^−10^ S cm^−1^ for the TpPaSO_3_H COF, and 1.53 × 10^−10^ S cm^−1^ for the TpPa COF, respectively, while after the incorporation of the COFs and the PIL, all the PIL@COF composite membranes exhibited an improved proton conductivity within the whole studied temperature range (Figure 5e). Among the three PIL@COF composite membranes, PIL@TpBD(SO_3_H)_2_ achieved the highest proton conductivity of 3.20 × 10^−3^ S cm^−1^ at 90 °C, which was higher than that of PIL@TpPaSO_3_H (8.80 × 10^−4^ S cm^−1^) and PIL@TpPa (7.80 × 10^−4^ S cm^−1^). The enhancement in ionic conductivity for the PIL@COF composite membranes can be ascribed to the confinement effect of the rigid and ordered 1D nanoporous proton transport channels constructed in the 2D COFs. Especially for PIL@TpPaSO_3_H and PIL@TpBD(SO_3_H)_2_, the methanesulfonate counter-ion in the PIL acted as the intrinsic proton source and the –SO_3_H groups on COF nanochannels worked as the extrinsic proton source, resulting in a large amount of mobilizable protons. Meanwhile, the electrostatic interactions between the zwitterionic structure in the PIL and the –SO_3_H groups in the COFs further promoted proton dissociation and enabled efficient proton transporting between the negative sites. Within the spatial confinement of the 2D COF nanopores, the TpBD(SO_3_H)_2_ COF with the highest density of –SO_3_H groups and strongest electrostatic interactions with the PIL provided the most efficient proton-conducting pathways, resulting in the highest proton conductivity. Furthermore, the proton conductivity variations with the relative humidity (RH) of the PIL and PIL@COFs were evaluated, and are presented in Figure S14. All the PIL@COFs and pristine PIL membranes exhibited an improved proton conductivity with increasing RH, revealing that conductivity is highly dependent on water molecule-mediated hydrogen-bonding networks. The stabilities of the PIL@COF composite membranes were evaluated by the temperature-dependent conductivity and weight loss of PIL@TpBD(SO_3_H)_2_ under 90 °C and anhydrous conditions. As shown in Figure S15, PIL@TpBD(SO_3_H)_2_ can be stable and maintain its proton conductivity and weight within 60 h, which can be ascribed to the stable interfacial binding between the PIL and COF and its high structural stability. Meanwhile, the high density of –SO_3_H groups on the COF nanochannels as anchoring sites effectively confine the PIL inside the channels, thus avoiding the leakage of PIL and a decrease in proton conductivity.

The activation energy (*E*a) value was used to ascertain the proton conduction mechanism. A low activation energy value means a low barrier for proton transfer. The *E*a values of the PIL and PIL@COF composite membranes were obtained by fitting their temperature-related conductivities using the Arrhenius equation, derived from the slope values. As shown in Figure 5f, the *E*a value obtained for the PIL was calculated to be 1.70 eV. Upon the introduction of the spatial confinement of the TpPa COF, the *E*a value of the PIL@TpPa composite membrane decreased to 1.30 eV. Moreover, as the density of –SO_3_H groups within the nanochannels increased, the *E*a values of PIL@TpPaSO_3_H and PIL@TpBD(SO_3_H)_2_ were even further decreased to 1.14 eV and 1.05 eV, thereby indicating the construction of a more continuous conductive pathway to promote proton conduction within the composite membrane.

**Figure 6:**
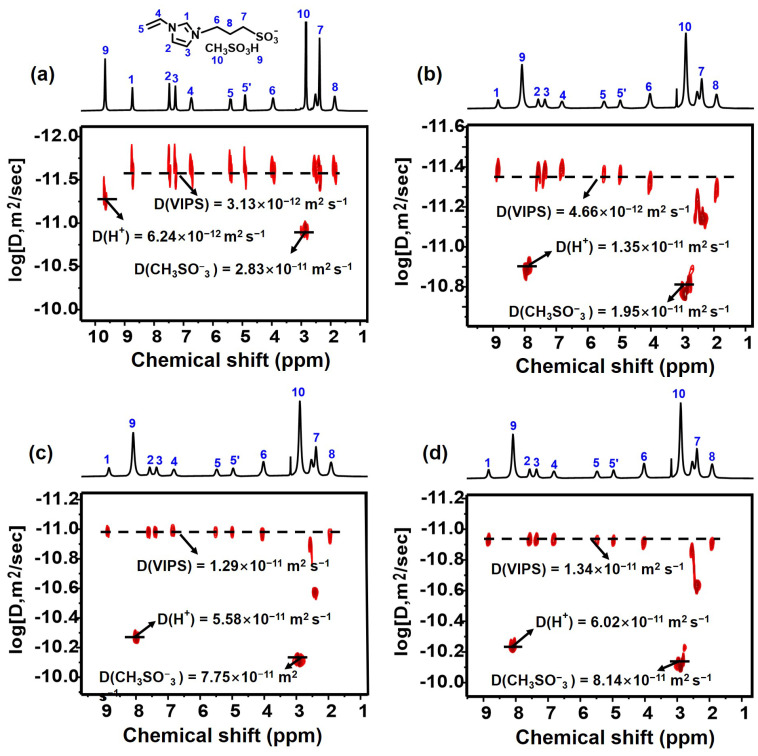
^1^H NMR DOSY spectra at 298 K for (**a**) PIL; (**b**) PIL@TpPa; (**c**) PIL@TpPaSO_3_H; (**d**) PIL@TpBD(SO_3_H)_2_.

To further evaluate the promotion effect of the spatial confinement of the COF nanochannels on proton conductivity [47], pulsed-field gradient nuclear magnetic resonance (PFG-NMR) was employed to investigate the diffusion coefficients and ion dissociation degrees of the pure PIL and the PIL within the 2D COF-confined system. Taking the PIL monomer as the model, diffusion-ordered spectroscopy (DOSY) measurements were performed at a frequency of 600 MHz in a coaxial NMR tube, with the inner tube containing DMSO-d6 and the outer tube containing either the PIL monomer or a mixture of the PIL monomer with a COF powder. The diffusion coefficients of the PIL monomer and the PIL monomer confined by COF nanochannels were obtained from the DOSY spectra (Figure 6a–d, Tables S1–S4). The diffusion coefficients of VIPS, [H^+^], and [CH_3_SO_3_^−^] in the bulk PIL monomer were 3.13 × 10^−12^, 6.24 × 10^−12^, and 2.83 × 10^−11^ m^2^ s^−1^, respectively. Due to the covalent combination of its cation and anion, the zwitterionic VIPS exhibited relatively low dynamics. The diffusion of protons was also limited, which can be attributed to the strong dipole interactions with the –SO_3_^−^ group in VIPS. For the PIL monomer confined by TpPa COF, the diffusion coefficients of VIPS, [H^+^], and [CH_3_SO_3_^−^] in PIL@TpPa were 4.66 × 10^−12^, 1.35 × 10^−11^, and 1.95 × 10^−11^ m^2^ s^−1^, respectively, which were all significantly larger than that of the pure PIL, while for the PIL confined by a COF with high-density –SO_3_H groups, the diffusion coefficients of [H^+^] increased to 5.58 × 10^−11^ m^2^ s^−1^ for PIL@TpPaSO_3_H and 6.02 × 10^−11^m^2^ s^−1^ for PIL@TpBD(SO_3_H)_2_, confirming the promotion of proton transport by the high-density and long-range order of sulfonic acid groups on the COF nanochannels. Transport number, which represents the relative contribution of a specific ion to the total ionic conductivity, is another fundamental parameter for evaluating proton migration behavior. Based on the diffusion coefficients obtained from the NMR DOSY measurements, the transport number of protons for the PIL and PIL@COFs can be calculated from Equation (3) [47]. The proton transport numbers for the PIL, PIL@TpPa, PIL@TpPaSO_3_H, and PIL@TpBD(SO_3_H)_2_ were calculated to be 0.18, 0.41, 0.42, and 0.43, respectively. Compared to the PIL, the relatively high proton transport numbers of the PIL@COFs are mainly due to the gating effect of the rigid and regular 1D nanoporous proton transfer channels of the COFs. Furthermore, the electrostatic interaction between the high-density –SO_3_H groups within the channels and the imidazole groups of the PIL promoted the dissociation of protons, resulting in an improved proton motion. In accordance with the EIS results, the protons in the COF-nanoconfined PIL can diffuse remarkably faster than the pure PIL, revealing that COFs, especially COFs with high-density –SO_3_H groups, can significantly improve the dynamic behaviors of protons. To further demonstrate the dissociation of protons, DFT-based calculations were performed using the Quickstep module of the CP2K package with the Gaussian plane wave (GPW) method. As shown in Figure S16, with the addition of VIPS, the bond length of the H-O bonds in the –SO_3_H groups in the TpBD(SO_3_H)_2_ COF increased from 0.98 Å to 1.10 Å, with the proton dissociation energy varying from −14.3 eV to −4.42 eV, demonstrating enhanced proton dissociation and transport.

## 3. Materials and Methods

### 3.1. Materials

Hexamethylene tetramine, acetone, and hydrochloric acid were purchased from Sinopharm Chemical Reagent Co (Shanghai, China). Phloroglucinol, p-phenylenediamine, methanesulphonic acid (PaSO_3_H), 2,5-diaminobenzenesulfoni acid, 4,4′-diaminodiphenyl ether-3,3′-disulfonic acid (BD(SO_3_H)_2_), 2-nitrobenzenesulfonyl chloride, and trifluoroacetic acid were all purchased from Meryer (Shanghai, China). 1-Vinylimidazole and 1,3-propanesultone were purchased from Macklin (Shanghai, China). 1,2-Dichlorobenzene, 1-butanol, 1,4-dioxane, and acetic acid (HOAc) were purchased from TCI (Gurugram, India). Tetrahydrofuran, methanol, dichloromethane, dimethyl sulfoxide, and dimethyl acetamide were purchased from Innochem (Pyeongtaek, Republic of Korea). Magnesium sulfate, diatomaceous earth, and sodium hydroxide were purchased from the Tansoole platform (Adamas (Shanghai, China)). Deionized water was used for all experiments.

### 3.2. Synthesis of 2,4,6-Triformylphloroglucinol (TFP)

Hexamethylenetetramine (HMT) (12.6 g, 90 mmol) and phloroglucinol (5.0 g, 40 mmol) were sequentially added to a 250 mL round-bottom flask under an inert atmosphere. Afterwards, 78 mL of CF_3_COOH was added to the mixture, and the resulting mixture was heated to reflux at 100 °C for 2 h. Subsequently, approximately 128 mL of 3 M hydrochloric acid was slowly added dropwise, and the solution was heated to reflux at 100 °C for 1.5 h. After being cooled to room temperature, the solution was filtered through diatomaceous earth. The filtrate was then extracted with dichloromethane at least three times. The organic phase was dried overnight over anhydrous magnesium sulfate and subsequently filtered. Finally, the solvent was removed under reduced pressure using a rotary evaporator, and the resulting product was vacuum dried at 60 °C to obtain a white powder with a yield of 15.7%. The ^1^H NMR and ^13^C NMR spectra of TFP are shown in Figure S1a and S1b, respectively.

### 3.3. Synthesis of 1-vinyl-3-(3-Sulfopropyl)-imidazolium Methanesulfonate

1-Vinylimidazole (9.41 g, 0.1 mol) was dissolved in 60 mL of acetone. Then, an equimolar amount of 1,3-propanesultone (12.24 g, 0.1 mol) dissolved in 40 mL of acetone was added to the above solution using a constant-pressure dropping funnel. The mixture was stirred under a nitrogen atmosphere at 0 °C for 2 h, then warmed up to room temperature with stirring for 5 days. After the reaction was completed, a white solid was obtained by vacuum filtration. The precursor was washed with acetone at least three times, and dried under vacuum conditions at room temperature to give a white solid, 1-vinyl-3-(3-sulfopropyl)-imidazolium (VIPS). The ^1^H NMR and ^13^C NMR spectra of VIPS are shown in Figure S2a and S2b, respectively.

Next, the obtained VIPS (2.16 g, 0.1 mol) was dissolved in a small amount of methanol. An equimolar amount of methanesulfonic acid (0.96 g, 0.1 mol) was added to the above solution. The mixture was stirred at room temperature for 24 h. The resulting liquid was dried under vacuum at room temperature for 5 h to obtain the PIL monomer (1-vinyl-3-(3-sulfopropyl)-imidazolium methanesulfonate) with a yield of 95%.

### 3.4. Synthesis of TpPa COF, TpPaSO_3_H COF and TpBD(SO_3_H)_2_ COF

#### 3.4.1. Synthesis of TpBD (SO_3_H)_2_ COF

TFP (21.0 mg, 0.10 mmol) and (BD(SO_3_H)_2_) (51.6 mg, 0.15 mmol) were added to a Pyrex tube. Then, a 1,4-dioxane/homotrimethylbenzene solvent mixture (1/4 (*v*/*v*), 1.2 mL) and 6 M HOAc (0.2 mL) were added to the Pyrex tube. The mixture was sonicated for 20 min. The reaction tube was subjected to three freeze–pump–thaw cycles to remove gases. The tube was then sealed and placed in an oven at 120 °C for 3 days. Afterward, the substance was gathered and rinsed with dimethylacetamide, dimethyl sulfoxide, ion-exchanged water, and dry tetrahydrofuran to eliminate any remaining monomers and oligomers. The TpBD (SO_3_H)_2_ COF was dried under vacuum at 120 °C overnight, producing a deep red powder with a yield of 53%.

#### 3.4.2. Synthesis of TpPaSO_3_H COF

TFP (21.0 mg, 0.10 mmol) and PaSO_3_H (28.1 mg, 0.15 mmol) were added to a Pyrex tube. Then, an o-dichlorobenzene/butanol solvent mixture (1/1(*v*/*v*), 1.0 mL) and 6 M HOAc (0.2 mL) were added to the Pyrex tube. The mixture was sonicated for 10 min. The reaction tube was subjected to three freeze–pump–thaw cycles to remove gases. The tube was then sealed and placed in an oven at 120 °C for 3 days. Afterwards, the product was collected and washed with ion-exchanged water and anhydrous tetrahydrofuran to remove the remaining oligomers and monomers. The TpPaSO_3_H COF was dried under vacuum at 120 °C overnight, resulting in a maroon powder with a yield of 83%.

#### 3.4.3. Synthesis of TpPa COF

TFP (21.0 mg, 0.10 mmol) and p-phenylenediamine (16.8 mg, 0.15 mmol) were added to a Pyrex tube. Then, an o-dichlorobenzene/butanol solvent mixture (1/1(*v*/*v*), 1.0 mL) and 3M HOAc (0.2 mL) were added to the Pyrex tube. The mixture was sonicated for 10 min. The reaction tube was subjected to three freeze–pump–thaw cycles to remove gases. The tube was then sealed and placed in an oven at 120 °C for 3 days. Afterwards, the product was collected and washed with ion-exchanged water and anhydrous tetrahydrofuran to remove residual monomers and oligomers. The TpPa COF was desiccated under vacuum at 120 °C for the duration of the night, resulting in a maroon powder with a yield of 85%.

### 3.5. Production of PIL, PIL@TpPa, PIL@TpPaSO_3_H, and PIL@TpBD(SO_3_H)_2_ Composite Membranes

PIL monomers (31.2 mg, 0.1 mmol) were mixed with a certain amount of ethylene glycol dimethacrylate (5 wt% by mass of PIL monomers) as a cross-linker and 2-hydroxy-2-methylpropiophenone (2 wt% by mass of PIL monomers) as an initiator. The solution was stirred for 24 h under light-shielding conditions. Then, the mixture was placed between two quartz substrates, and the thickness of the sample was controlled by changing the distance of quartz substrates with silica spacers. After that, the sandwiched mixture between the two quartz substrates was irradiated with a UV arc lamp at an intensity of 9 mW/cm^2^ for 30 min at room temperature. To ensure the maximum conversion, the reaction was illuminated at both the top and bottom surfaces of the substrates to yield the PIL film.

For the preparation of the PIL@COF composite membranes, 0.75 mmol COF powders (i.e., 19.6 mg TpPa, 25.7 mg TpPaSO_3_H, and 37.4 mg TpBD(SO_3_H)_2_) were comminuted and dried under vacuum conditions at 120 °C for 12 h to remove impurities and moisture. Afterwards, the pre-heated COF powders were separately mixed with the PIL monomer (31.2 mg, 0.1 mmol), 5 wt% ethylene glycol dimethacrylate as a cross-linker, and 2 wt% 2-hydroxy-2-methylpropiophenone as an initiator. The subsequent photopolymerization processes were similar to those of the PIL film into PIL@TpPa, PIL@TpPaSO_3_H, and the PIL@TpBD(SO_3_H)_2_ membrane, respectively (approximately 9 mm in diameter, about 1 mm in thickness). The corresponding photographs of the PIL@COF composite membranes are shown in Figure S3.

### 3.6. Proton Conductivity Measurements

The conductivity of the membranes was measured using a CHI760D electrochemical workstation (Shanghai Chenhua, China) by a two-electrode alternating current impedance method. The membrane samples were first dried under vacuum conditions at 100 °C to remove impurities and moisture, and then placed between two stainless steel electrodes to form a coin cell configuration in the glovebox under an Ar atmosphere. Prior to taking measurements, the cells were put into an oven and allowed to stabilize for 1 h at different temperatures. During conductivity testing, a frequency range of 1 to 106 Hz was utilized, with an oscillation voltage of 5 mV. The membrane resistances were determined by analyzing the Nyquist curves obtained from the measurements.

The proton conductivity (σ, S cm^−1^) of the PIL and PIL@COF was estimated using the following equation:

(1) *σ* = *L*/(*RA*)

where *σ* represents the proton conductivity in S cm^−1^, *L* is the thickness of the pellets in cm, *A* is the surface area of the pellets in cm^2^, and *R* is the low intercept of the high-frequency semi-circle on a complex impedance plane with the Z axis (Ω). To fit the data, an equivalent circuit (Figure S4) was adopted and analyzed using the ZView software (Version 3.5.0.10).

The Arrhenius kinship between proton conductivity (*σ*) and temperature (*T*) can be expressed as follows:

(2) ln(*σ*) = ln(*σ*_0_) − (*E*a/*RT*)

where *σ* represents the conductivity, *σ*_0_ represents the pre-exponential factor, *E*a represents the activation energy of proton conduction, *T* represents the absolute temperature in Kelvin, and *R* represents the gas constant (8.314 J K^−1^ mol^−1^).

### 3.7. Diffusion Coefficient Measurements

To explore the diffusion of the PIL monomers, diffusion-ordered spectroscopy (DOSY) measurements were conducted at a frequency of 600 MHz using pulsed-field gradient nuclear magnetic resonance (PFG-NMR). The tests were performed in a coaxial NMR tube, with the inner tube containing DMSO-d6 and the outer tube containing either the PIL monomer or a mixture of the PIL monomer with COF powder. The diffusion coefficients of the PIL monomer and the PIL monomer confined by 2D COF nanochannels were obtained from the NMR data. The transport number of protons was calculated using the following equation:

(3) $$t_{H^{+}}=\frac{D_{H^{+}}}{D_{{\mathrm{CH}}_{3}{\mathrm{SO}}_{3}^{-}}+D_{H^{+}}}$$

where $t_{H^{+}}$ is the transport number of H^+^, $D_{H^{+}}$ is the diffusion coefficient of H^+^, and $D_{{\mathrm{CH}}_{3}{\mathrm{SO}}_{3}^{-}}$ is the diffusion coefficient of CH_3_SO_3_^−^.

### 3.8. Characterization

Thermogravimetric analysis (TGA) was performed using a TL 9000 thermogravimetric analyzer under a nitrogen atmosphere between 30 and 700 °C with a heating rate of 10 °C min^−1^. Raman spectra were obtained using a Reflex inVia Raman spectrometer and a 514 nm laser. ^1^H NMR spectra and ^13^C NMR spectra were recorded using a Bruker AVANCE NEO 400 MHz spectrometer with DMSO-d_6_, D_2_O, or CDCl_3_ as solvents. The CuKax beam source was used to collect powder X-ray diffraction (PXRD) data in the range of 2° to 35° at a scan speed of 5° min^−1^. Fourier transform infrared spectroscopy (FT-IR) was carried out using an FT/IR-6800 in the range of 400–4000 cm^−1^. The nitrogen adsorption isotherm method was used to calculate the specific surface area by the Brunauer–Emmett–Teller (BET) method, using BELSORP MAX II at 77 K. The pore size distribution was evaluated using the non-local density flood theory (NLDFT) method. Scanning electron microscopy (SEM) was carried out using a Hitachi Regulus Model 8100 instrument for imaging. X-ray photoelectron spectroscopy (XPS) was carried out using a Thermo Fisher spectrometer. Differential scanning calorimetry (DSC) measurements were carried out using the Swiss Mettler DSC3 differential scanning calorimeter. Electrochemical tests were performed on a CHI 760 electrochemical workstation.

## 4. Conclusions

In summary, composite proton-conducting membranes were fabricated by the incorporation of a proton-containing PIL as the intrinsic proton supplier and two-dimensional (2D) covalent organic frameworks (COFs) bearing –SO_3_H groups as the extrinsic proton source via in situ polymerization. As expected, the highly ordered configuration of the COFs provided an effective pathway for efficient proton transport. The migration of protons in nanoconfined PIL (PIL@COF) has been demonstrated to be remarkably promoted compared with pure PIL. Benefiting from the highest density of –SO_3_H groups on the long-range ordered nanochannels of the TpBD(SO_3_H)_2_ COF and the enhanced electrostatic interactions with PIL, the as-synthesized PIL@TpBD(SO_3_H)_2_ exhibited a comparable proton conductivity of 3.20 × 10^−3^ S cm^−1^ at 90 °C. ^1^H NMR DOSY spectra revealed that the diffusion and dissociation of protons were drastically promoted upon nanoconfinement, demonstrating the promising efficiency of nanochannels in proton conduction. Moreover, all the obtained PIL@COF composite membranes possessed outstanding mechanical and thermal stability, which is crucial for their practical application. This work provides a rational structural guideline and methodology for the design of next-generation ion-conducting materials.

## Data Availability

Data are contained within the article and Supplementary Materials.

## Supplementary Materials

The following supporting information can be downloaded at: https://www.mdpi.com/article/10.3390/molecules30051004/s1, Figure S1–S16 and Tables S1–S4: ^1^H NMR and ^13^C NMR spectra of monomers; Photographs of PIL and PIL@COF composite membranes; DSC curves of PIL and PIL@COFs; FTIR spectra of TpBD(SO_3_H)_2_ COF and corresponding monomers; XPS spectra of PIL, COFs, and PIL@COFs; EDS mapping images of PIL and PIL@COFs; Conductivities of COFs; Chemical shift and error of diffusion coefficients from ^1^H DOSY NMR.
