# Supplementary material for: The Incorporation of Nanoconfined Poly(ionic liquid)s with Two-Dimensional Covalent Organic Frameworks to Enhance Proton Conduction
Source: Molecules. 2025 Feb 21;30(5):1004. doi: 10.3390/molecules30051004 (PMC11901798; doi:10.3390/molecules30051004)
Supplement: Supplementary file 1 [file molecules-30-01004-s001.zip › molecules-3428035-supplementary.pdf]

Supplementary Information

The Incorporation of Nanoconfined Poly(ionic liquid)s with Two-Dimensional Covalent Organic Frameworks to Enhance Proton Conduction

Yonghong Wang, Xiaoxiao Liang, Ming Wang, Jiahui Wang, Yanan Gao * and Fei Lu *

Key Laboratory of Ministry of Education for Advanced Materials in Tropical Island Resources, School of Chemistry and Chemical Engineering, Hainan University, Haikou 570228, China

*   Correspondence: ygao@hainanu.edu.cn (Y.G.); lufei@hainanu.edu.cn (F.L.)

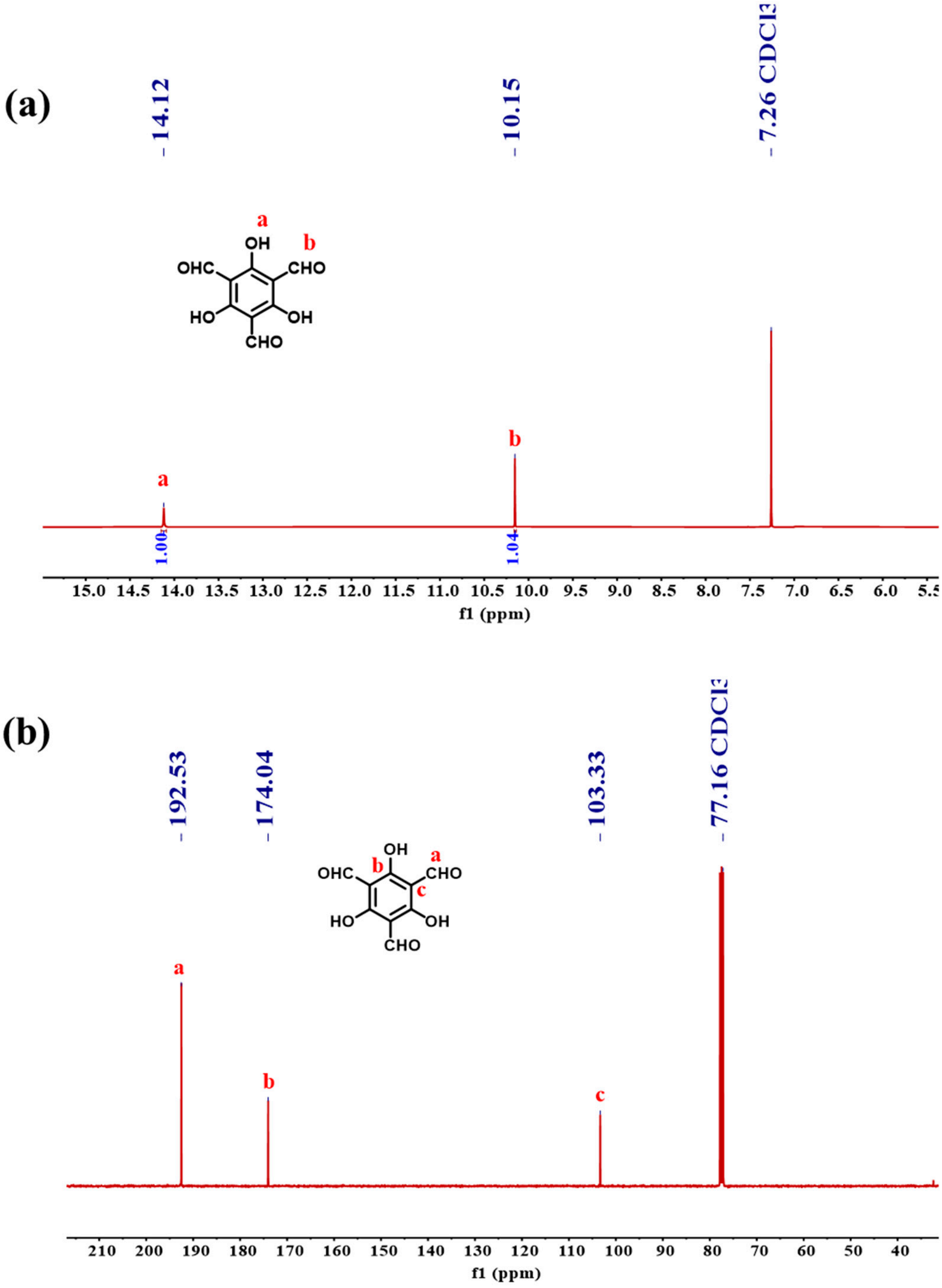

Figure S1. (a) ^{1}H NMR and (b) ^{13}C NMR spectra of 2,4,6-triformylphloroglucinol (TFP).

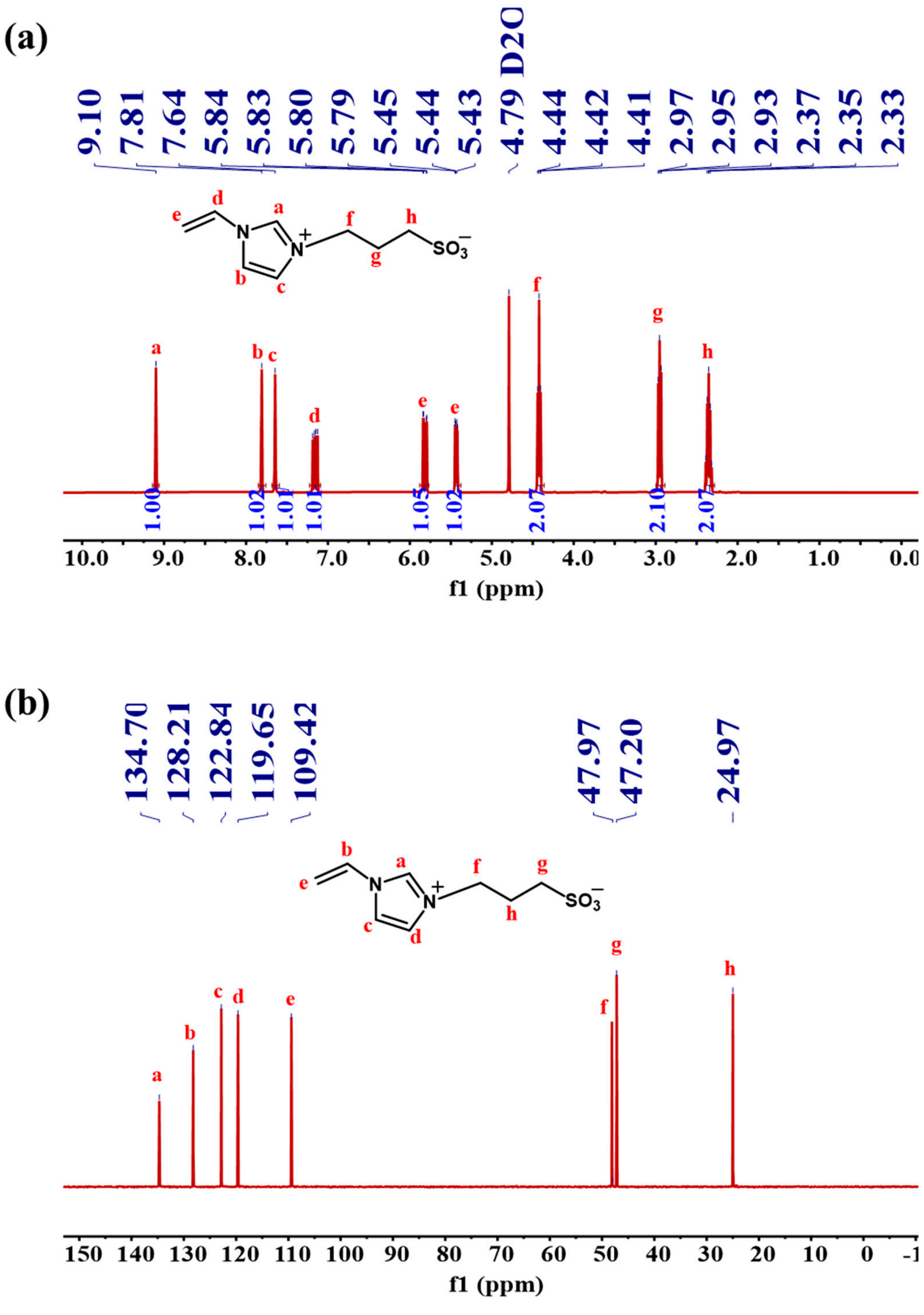

Figure S2. (a) ^{1}H NMR and (b) ^{13}C NMR spectra of 3-(1-vinyl-3-imidazolyl) propanesulfonate (VIPS).

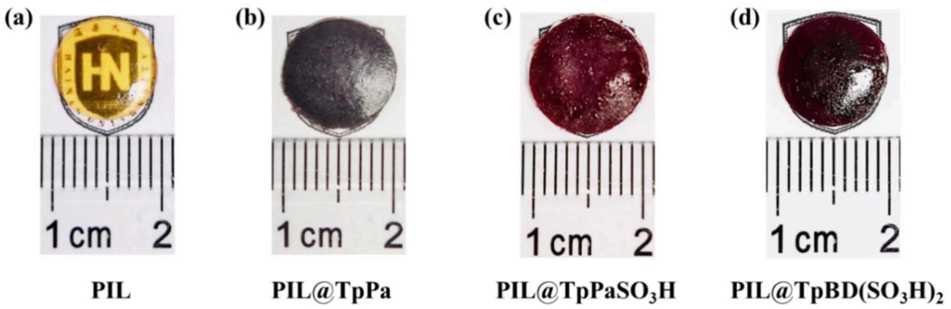

Figure S3. Photographs of the PIL and PIL@COF composite membranes for proton conductivity measurements.

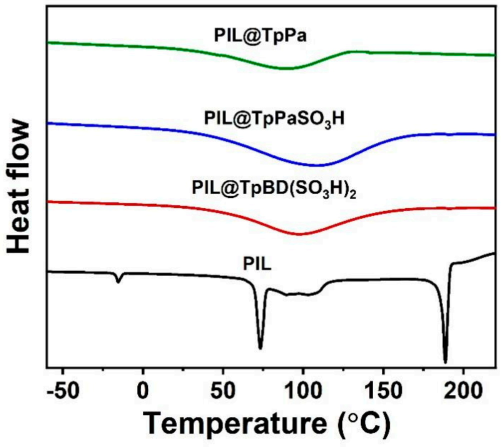

Figure S4. DSC curves of PILand PIL@COF.

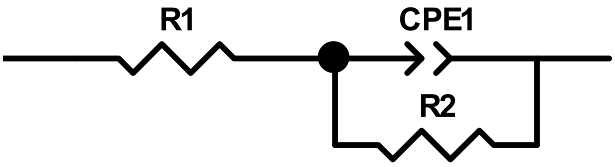

Figure S5. Equivalent circuit model representation for the proton conduction in PIL@COFs and PIL wherein R1, R2, represent resistors and CPE1 represent imperfect capacitors in the circuit.

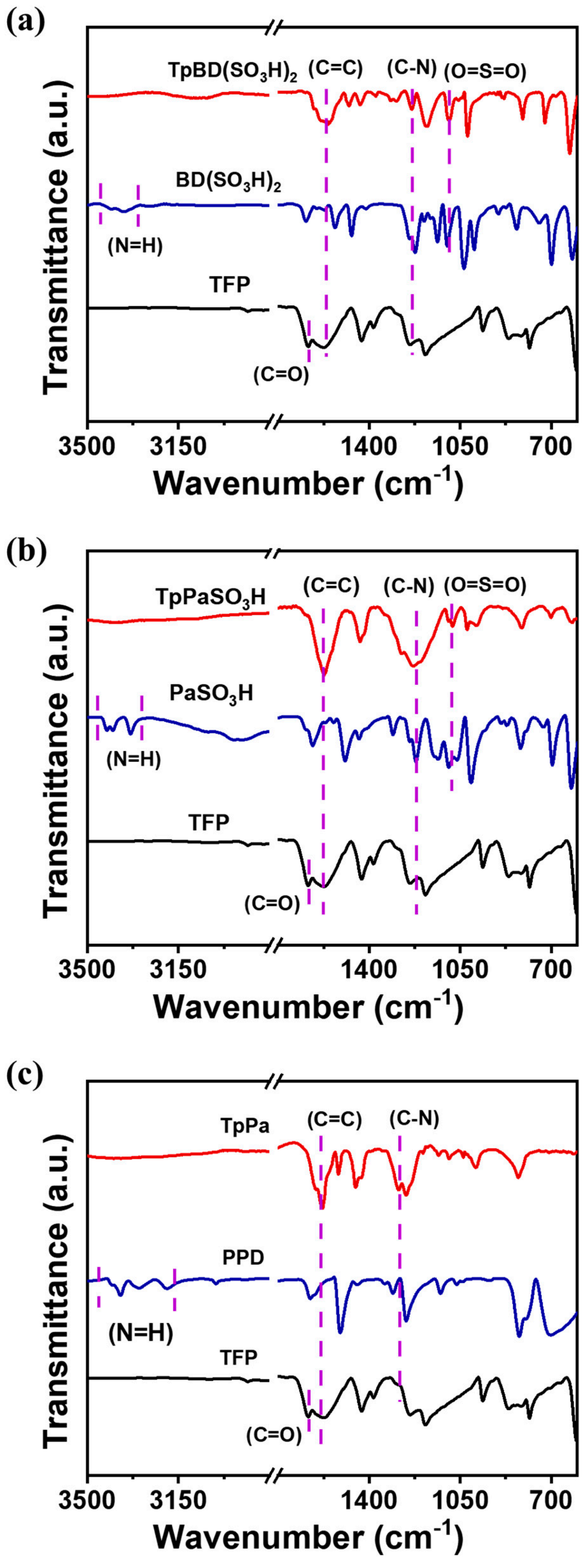

Figure S6. (a) FTIR spectra of $TpBD(SO_3H)_2$ COF and corresponding monomers. (b) FTIR spectra of $TpPaSO_3H$ COF and corresponding monomers. (c) FTIR spectra of TpPa COF and corresponding monomers.

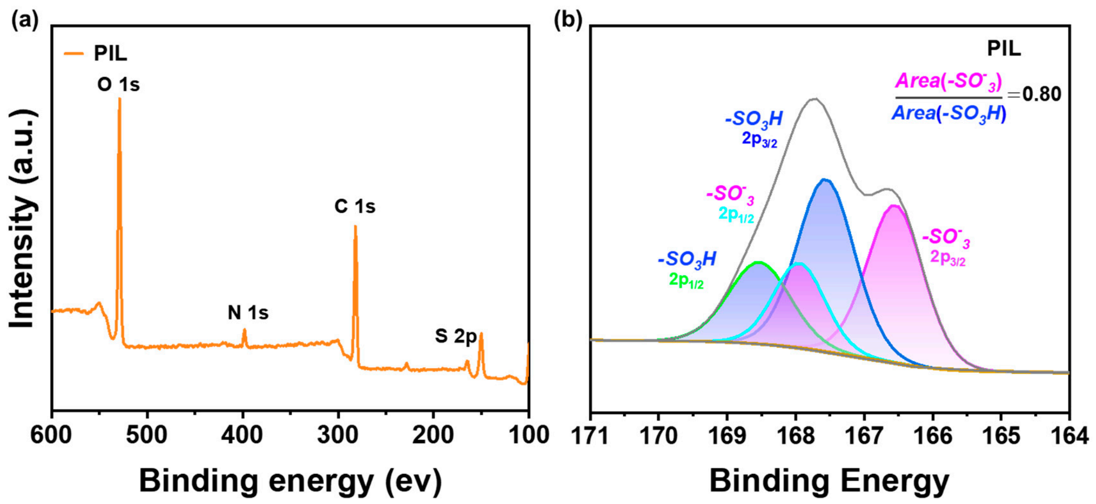

Figure S7. (a) XPS spectra of PIL. (b) S2p XPS spectra of PIL.

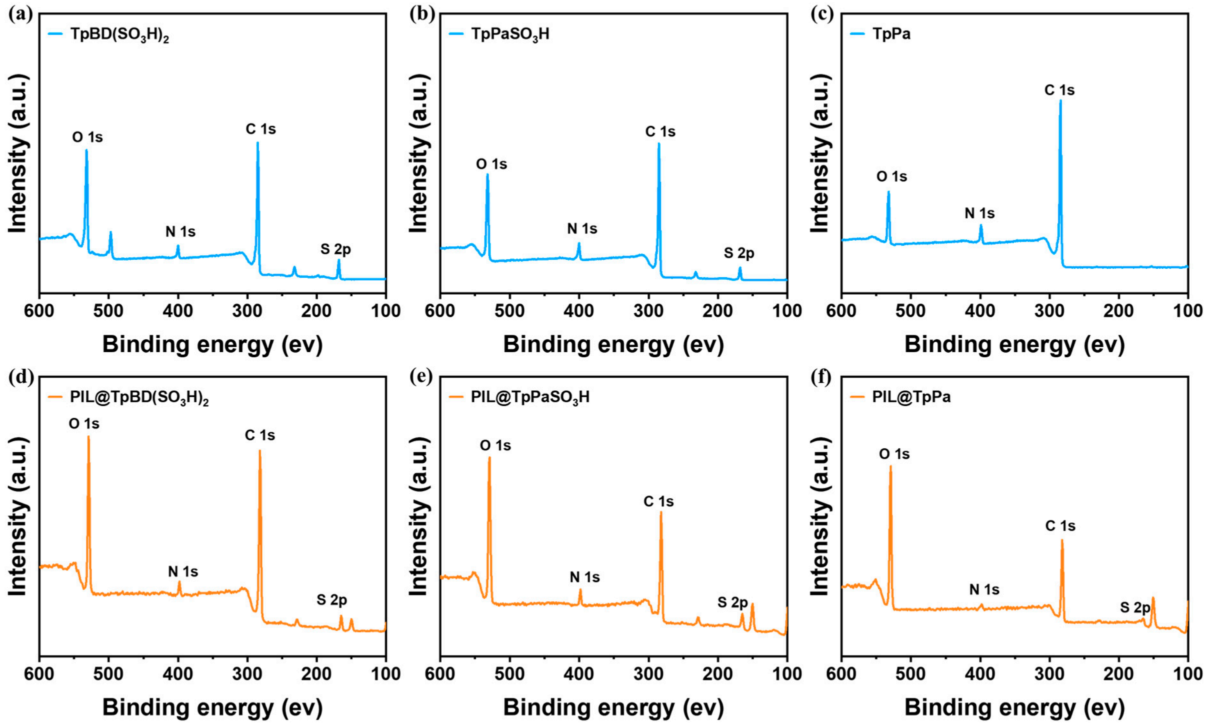

Figure S8. XPS spectra of COFs and PIL@COF.

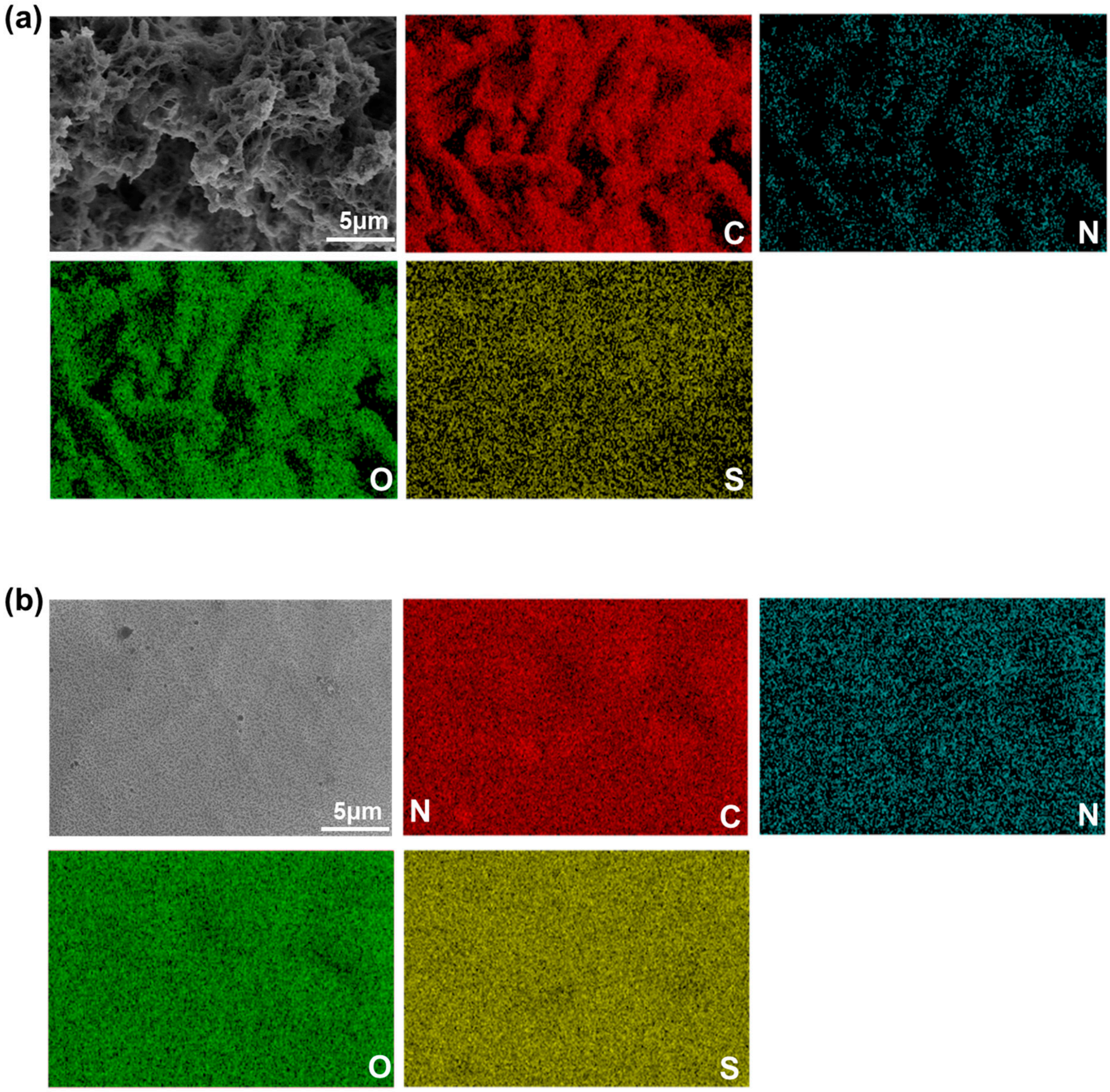

Figure S9. EDS mapping images of (a) TpBD(SO_3H)$_2$ COF and (b) PIL@TpBD(SO_3H)$_2$.

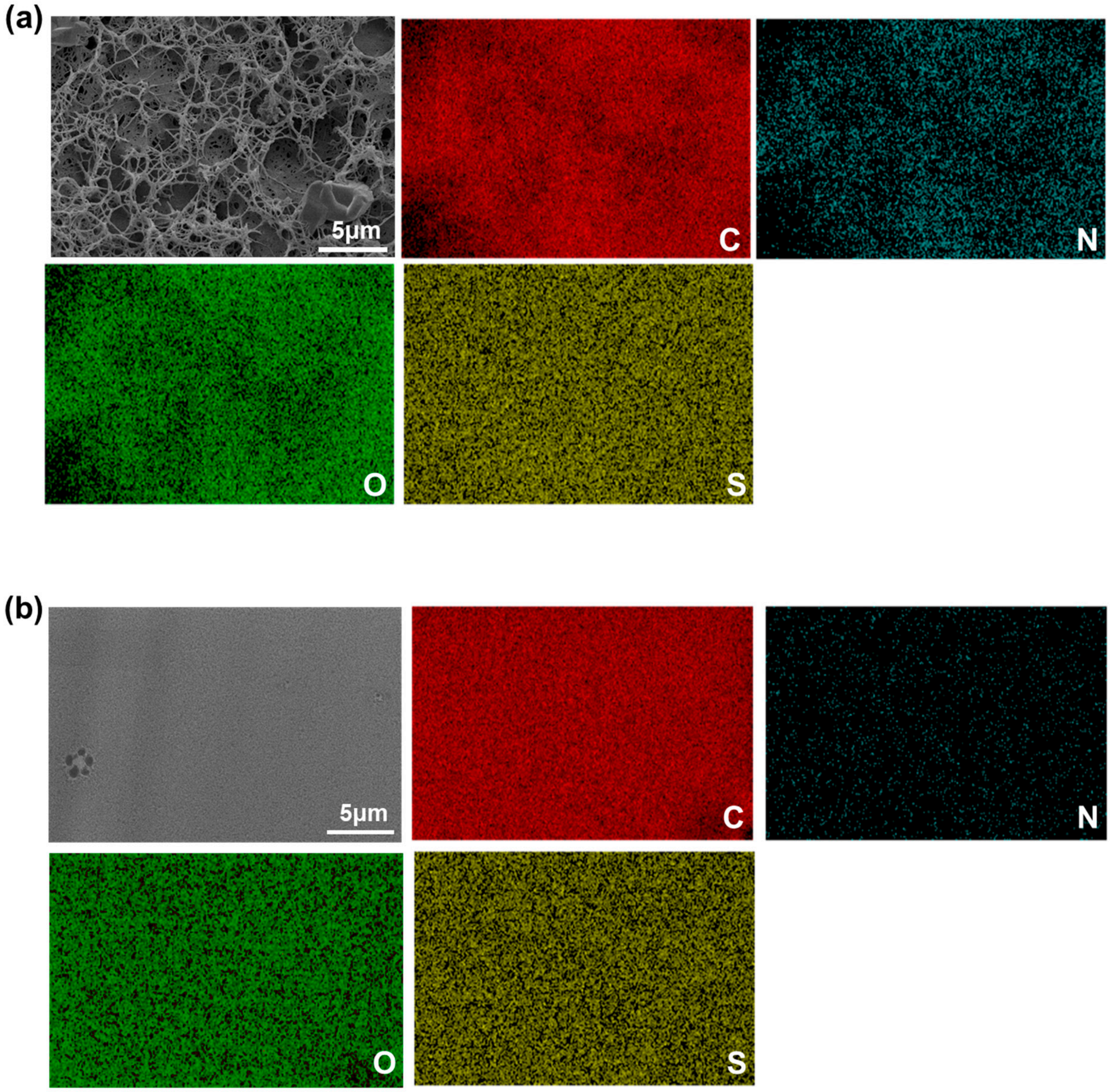

Figure S10. EDS mapping images of (a) $TpPaSO_3H$ COF and (b) PIL@$TpPaSO_3H$.

(a)

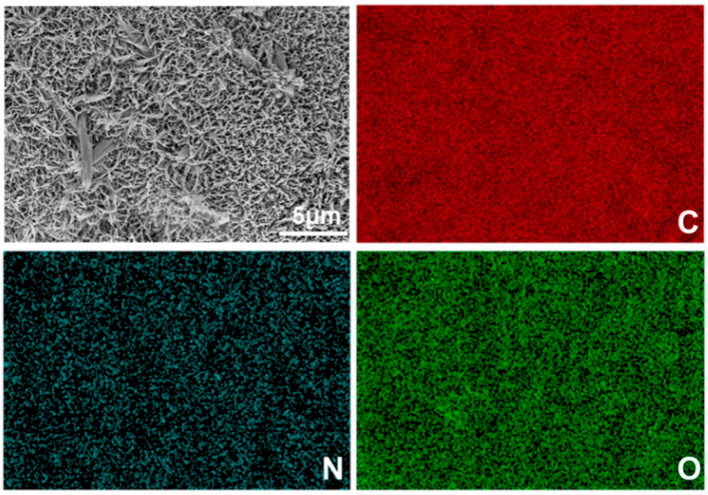

(b)

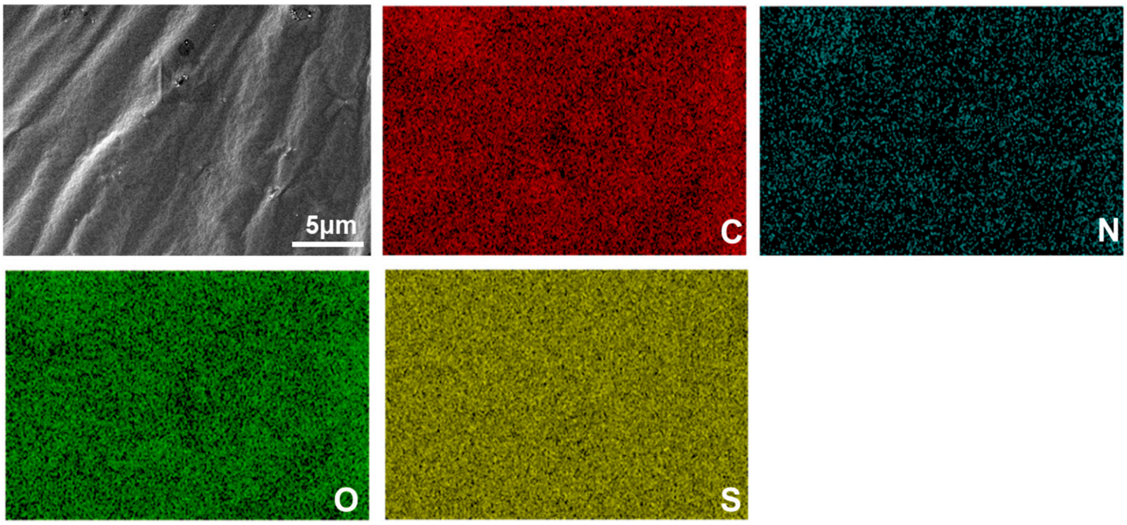

Figure S11. EDS mapping images of (a) TpPa COF and (b) PIL@TpPa.

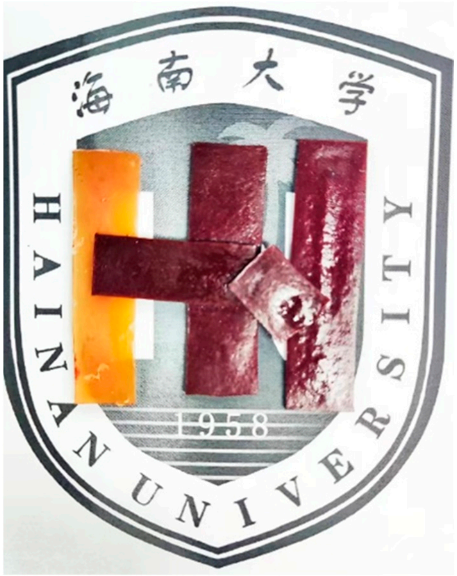

Figure S12. Photographs of highly flexible PIL and PIL@COFs.

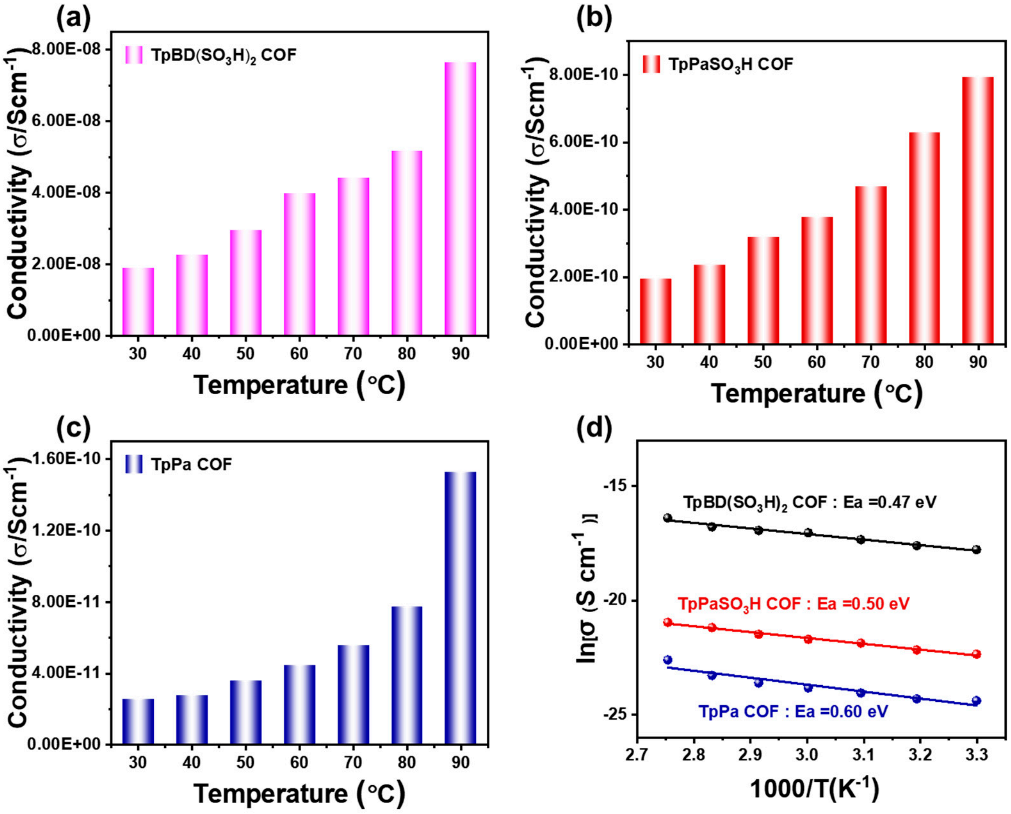

Figure S13. The conductivities of (a) TpBD(SO_3H)$_2$ COF, (b) TpPaSO_3H COF and (c) TpPa COF in the temperature range of 30℃ to 90 ℃. (d) Arrhenius plots for TpBD(SO_3H)$_2$ COF, TpPaSO_3H COF and TpPa COF.

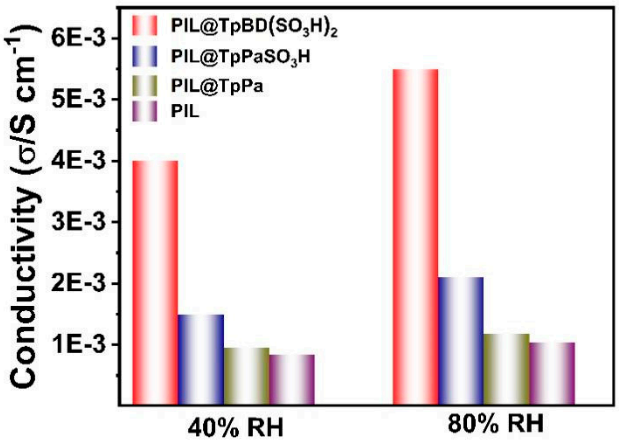

Figure S14. RH-dependent conductivities of PIL and PIL@COF under at 90 °C.

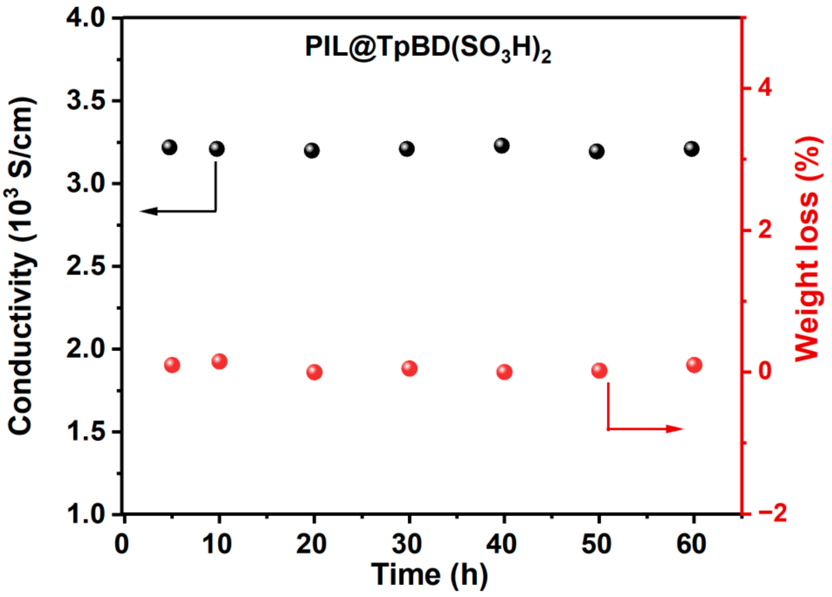

Figure S15. Temperature-dependent conductivity and weight loss of

PIL@TpBD(SO_3H)$_2$ under 90 °C and anhydrous conditions.

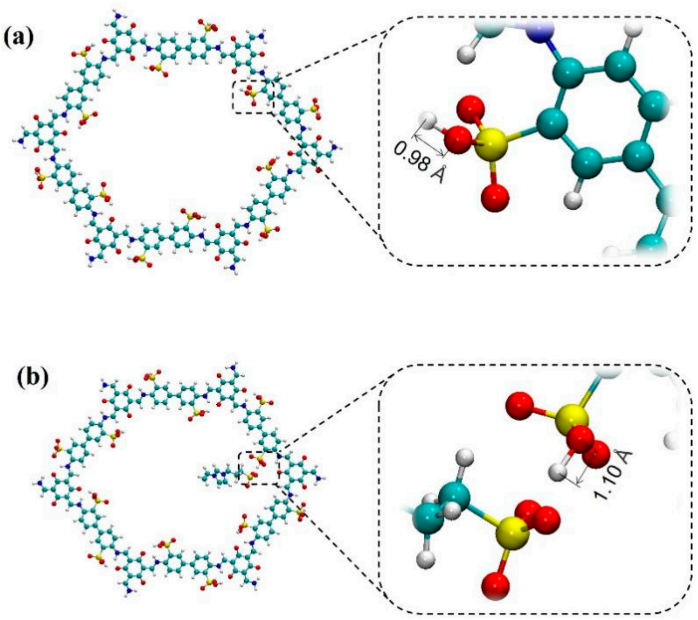

Figure S16. Theoretical elucidation of O-H bond in -SO_3H group for (a) pristine $TpBD(SO_3H)_2$ COF and (b) $TpBD(SO_3H)_2$ COF in the presence of VIPS.

Table S1. Chemical shift and error of diffusion coefficients for peaks of bulk PIL recorded from ^1H DOSY NMR at 298 K.

Peak name	Chemical Shift [ppm]	Diffusion Coefficient [m^2/s^{-1}]	Error
9	9.663	6.24e-12	1.199e-13
1	8.736	3.10e-12	7.269e-14
2	7.481	3.09e-12	7.030e-14
3	7.277	3.11e-12	7.384e-14
4	6.736	3.04e-12	5.384e-14
5	5.419	2.99e-12	9.187e-14
5‘	4.912	2.97e-12	5.504e-14
6	3.959	3.12e-12	6.499e-14
10	2.837	1.34e-11	2.929e-13
7	2.378	3.54e-12	7.713e-14
8	1.864	3.21e-12	6.817e-14

Table S2. Chemical shift and error of diffusion coefficients for peaks of bulk PIL@TpPa recorded from ^1H DOSY NMR at 298 K.

Peak name	Chemical Shift [ppm]	Diffusion Coefficient [m^2/s^{-1}]	Error
1	8.856	4.01e-12	1.960e-13
9	7.927	1.35e-11	1.049e-12
2	7.583	4.09e-12	3.087e-13
3	7.372	4.08e-12	2.239e-13
4	6.826	3.97e-12	3.595e-13
5	5.495	4.38e-12	2.370e-13
5‘	4.977	4.37e-12	2.114e-13
6	4.032	4.84e-12	3.016e-13
10	2.892	1.95e-11	1.257e-12
7	2.377	7.01e-12	2.931e-13
8	1.920	5.23e-12	2.927e-13

Table S3. Chemical shift and error of diffusion coefficients for peaks of bulk PIL@TpPaSO_3H recorded from ^{1}H DOSY NMR at 298 K.

Peak name	Chemical Shift [ppm]	Diffusion Coefficient [m^2/s^{-1}]	Error
1	8.849	1.18e-11	1.822e-13
9	8.093	6.02e-11	2.537e-12
2	7.577	1.17e-11	1.595e-13
3	7.366	1.17e-11	1.851e-13
4	6.825	1.17e-11	2.140e-13
5	5.495	1.20e-11	3.894e-13
5‘	4.976	1.22e-11	2.760e-13
6	4.032	1.25e-11	3.589e-13
10	2.896	8.14e-11	1.913e-12
7	2.393	2.40e-11	3.797e-13
8	1.924	1.28e-11	4.196e-13

Table S4. Chemical shift and error of diffusion coefficients for peaks of bulk PIL@TpBD(SO_3H)$_2$ recorded from ^{1}H DOSY NMR at 298 K.

Peak name	Chemical Shift [ppm]	Diffusion Coefficient [m^2/s^{-1}]	Error
1	8.882	1.03e-11	1.854e-13
9	7.992	5.58e-11	2.583e-12
2	7.607	1.04e-11	1.978e-13
3	7.394	1.04e-11	1.992e-13
4	6.853	1.03e-11	1.739e-13
5	5.536	1.05e-11	2.031e-13
5‘	5.018	1.05e-11	1.958e-13
6	4.060	1.11e-11	3.488e-13
10	2.918	7.75e-11	1.068e-12
7	2.418	2.70e-11	1.340e-12
8	1.9496	1.52e-11	3.196e-13
